# Promoter Region and Regulatory Elements of IGF and VIP Genes Associated With Reproductive Traits in Chicken

**DOI:** 10.1155/ijog/5574292

**Published:** 2025-05-26

**Authors:** Bosenu Abera, Hunduma Dinka, Hailu Dadi, Habtamu Abera

**Affiliations:** ^1^Department of Applied Biology, School of Applied Natural Science, Adama Science and Technology University, Adama, Ethiopia; ^2^Bio and Emerging Technology Institute, Addis Ababa, Ethiopia; ^3^Ministry of Innovation and Technology, Addis Ababa, Ethiopia

**Keywords:** CpG islands, IGF gene, promoter region, reproductive traits, tandem repeats, transcription factor, VIP gene

## Abstract

This study investigates the promoter region and regulatory elements of chicken insulin-like growth factor (IGF) and vasoactive intestinal polypeptide (VIP) genes associated with reproductive traits. Several in silico tools, such as Neural Network Promoter Prediction (NNPP), Multiple Expectation maximizations for Motif Elicitation (MEME-Suite), GC-Profiles, microsatellite prediction (MISA-web), CLC Genomics, Multiple Association Network Integration Algorithm (GeneMANIA), and Gene Ontology for Motifs (GOMO), were used to characterize the promoter regions and regulatory elements of IGF and VIP genes. The in silico analysis showed that the highest promoter prediction scores (1.0) for TSS were obtained for three gene sequences (IGFP4, VIP, and VIPR1), while the lowest promoter prediction score (0.8) was obtained for IGF1. The present analysis revealed that the best common motif, Motif II, resembles three major transcription factor families: zinc finger family, homeobox transcription factor family, and high-mobility group factor family, accounting for about 79.17%. This study found that 62.5% of the candidate transcription factors have interaction with the Wnt signalling pathway to regulate transcription. Key regulatory elements identified in this study, such as CPEB1, MAFB, SOX15, TCF7L2, TCF3, and TCF7, play critical roles in activating and repressing transcription, with significant implications for embryonic and nervous system development. In the current study, very rich CpG islands were identified in the gene body and promoter regions of IGF and VIP genes. Generally, in silico analysis of gene promoter regions and regulatory elements in IGF and VIP genes can be helpful for comprehending regulatory networks and gene expression patterns in promoter regions, which will guide new experimental studies in gene expression assays.

## 1. Introduction

The chicken insulin-like growth factor (IGF) and vasoactive intestinal peptide (VIP) genes are physiological candidates associated with reproductive traits [1, 2]. These genes are evolutionarily well-conserved peptides with sequence similarity among animals, including chickens, and play a key role in regulating signalling pathways in different animals [3]. The IGF system comprises IGF1 and IGF2 (Insulin-Like Growth Factor 2), along with their receptors IGF1R and IGF2R, IGF-Binding Proteins 1–6 (IGFBP1–6), and IGF2-Binding Proteins 1–3 (IGF2BP1–3). The VIP gene possesses two significant receptors, VPAC1 and VPAC2, which are extensively present in the central nervous system and peripheral tissues [4]. The biological functions of IGFs and the VIP gene are realized through their interaction with receptors [5–7]. The IGFs function as the major mediator of growth hormone (GH)–stimulated somatic growth [8], whereas the VIP gene regulates several ovarian functions such as follicular development, ovulation, steroidogenesis, and granulosa cell apoptosis [9].

Studies revealed that the process of chicken reproduction is regulated by the hypothalamic-pituitary-gonad axis [10]. Those findings imply that there are numerous genes associated with reproductive traits that are affecting the performance of chicken. Among others, the IGF systems and VIP gene are expressed in various organs in early embryonic developmental stages and play important fundamental roles in chicken embryo development [11, 12]. These genes also affect the reproductive traits of chicken via influencing gonadotropin-releasing hormone secretion [13–18]. Reproductive traits play a vital role in the genetic improvement of laying and broiler chickens. They dictate the fertility and feasibility of chicken production in the poultry industry [19]. Thus, there is a growing interest in identifying and characterizing specific genes and genomic regions that play a role in the regulation of reproductive processes.

Regulatory elements are DNA sequences that control gene expression through transcription initiation (promoters) and by enhancing transcription at distant regions (enhancers). Promoters are one of the key regulatory elements that belong to noncoding regions and determine the direction of transcription and regulate gene expression [20–22]. The promoter sequences are typically located directly upstream or at the 5⁣′ end of the transcription initiation site [23]. These regions contain CpG islands that are known to regulate gene expression through transcriptional silencing of the corresponding gene. About half of all CGIs self-evidently contain TSSs, as they coincide with promoters of annotated genes [24].

Studies on chickens identified many CpG islands and tissue-specific regulatory elements that could affect downstream genes [25–27]. Those findings indicate that an accurate identification of regulatory elements is fundamental for annotating genomes and understanding gene expression patterns. However, the regulatory mechanisms of these genes in the chicken reproduction traits have not yet been reported. In silico tools have been an integral part of the biological research designed to uncover meaningful information from biological data within a very short time. Therefore, the aim of this study was to predict promoter and regulatory elements of IGF and VIP genes associated with reproductive traits and thereby contribute to the improvement of reproductive performance and fertility in chickens.

## 2. Results

### 2.1. Identification of TSS and Promoter Regions of IGF and VIP Genes

The study of promoter regions for IGF and VIP genes associated with reproductive traits in chickens revealed a notable variation in the number of transcription start sites (TSSs), with 73.33% or 11 out of 15 sequences containing multiple TSSs (Table 1). Additionally, the study found that seven (46.7%) TSSs are located at a distance below −500 bp from the start codon. The TSSs were located in the upstream region ranging from −41 to −4626 bp (base pairs).

### 2.2. Common Candidate Motifs and Associated Transcription Factors in the Promoter Regions of IGF and VIP Genes

The current analysis discovered five binding motifs, of which two (I and III) were equally shared (73.3%) by all IGF and VIP genes in chicken, as shown in Table 2. Furthermore, Motif II was identified as the best common promoter motif for 80% of chicken IGF and VIP genes associated with chicken reproductive traits that serve as binding sites for TFs involved in the expression regulation of these genes.

**Figure 1:**
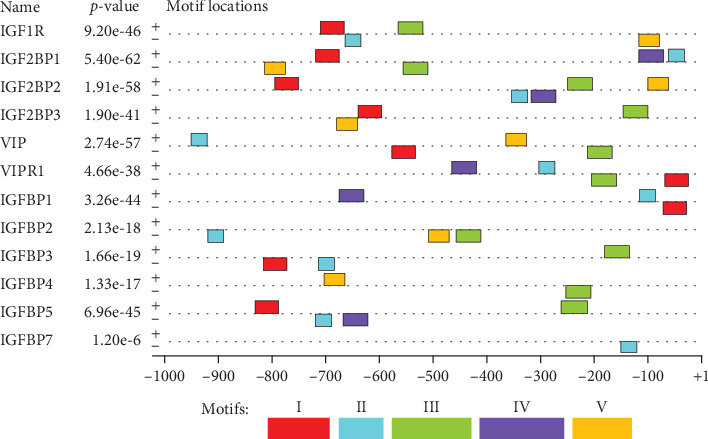
Block diagrams illustrating the positions of candidate motifs within the promoter region in relation to transcription start sites (TSSs).

In the present study, the majority (64.29%) of the candidate motifs were located and distributed between –800 and –200 bp, and the motifs were equally distributed in the positive and negative strands (Figure 1). The MEME search tool produced a sequence logo for Motif II, which is recognized as the most common motif to represent the information content. The TOMTOM web tool explored matched motifs for Motif II. As a result, Motif II matched with 24 known motifs found in databases (Table 3).

**Figure 2:**
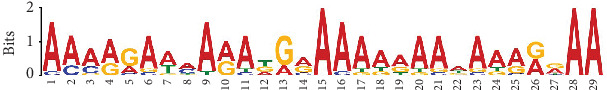
Sequence logos for Motif II, for promoter regions of IGF and VIP genes associated with reproductive traits in chicken [28].

The sequence logo for Motif II generated by MEME is presented in Figure 2. In this visualization, the size of each letter reflects how strongly that nucleotide is conserved at each position in the motif.

The current analysis indicates that the best common motif, Motif II, resembles three major transcription factor families: the zinc finger family, homeobox transcription factors, and high-mobility group domain factors, accounting for about 79.17% (19/24) (Table 3). This study also revealed that about 62.5% (15/24) of identified transcription factors were associated with the Wnt signalling pathway. The findings from the UniProt database show that candidate TFs such as CPEB1, MAFB, SOX15, TCF7L2, TCF3, and TCF7 are pivotal in activating or repressing transcription, playing essential roles in embryonic and nervous system development. Likewise, the transcription factor LEF1 is implicated in hair cell differentiation and follicle morphogenesis. The study also highlights that the Bcl11B transcription factors suppress transcription and are crucial in normal T-cell development, as identified from the UniProt database.

### 2.3. Investigation for CpG Islands and Tandem Repeats

CpG islands were investigated in the promoter and gene body regions of 12 IGFs and 3 VIP genes associated with reproductive traits in chicken using different computational tools. In this study, we analysed each gene promoter and discovered CpG islands in 11 out of the 15 promoters examined, with the exception of IGF1, IGF2, IGFBP1, and IGFBP7. Similarly, 13 CpG islands were identified within the gene body regions, as shown in Table 4. This indicated that IGF and VIP genes associated with chicken reproductive traits have very rich CGIs in their promoter and gene body regions. The highest length of CpG island was observed in the *VIP* gene promoter (2000 bp) showing similarity in length to the targeted query (2000 bp). Multiple CpG islands were observed in the promoter regions of the IGF2BP3, IGFBP3 (Insulin-Like Growth Factor–Binding Protein 3), and IGFBP4 (Insulin-Like Growth Factor–Binding Protein 4) genes, as well as in the body regions of the IGF1R, IGF2BP1, and IGF2BP2 genes (Table 4).

In order to investigate tandem repeats, 2 kb upstream promoter sequences of chicken IGF and VIP genes were used. Tandem repeats having one to four nucleotides were identified in the promoter regions of 10 IGFs and VIP genes and the gene body region of two genes (Table 5).

Analysis for CpG islands on both promoter region and gene body region using restriction enzyme *MspI* was also conducted (Tables 6 and 7). The in silico analysis results showed more CpG islands in gene body region compared to promoter region. In the present analysis, about 13 CGIs and 12 CGIs were found in gene body region and promoter region, respectively. The results indicated that chicken IGF and VIP genes were highly rich in CpG islands which is in agreement with the first GC-Profiles 2.0 searching tool.

### 2.4. Analysis of Gene–Gene Interactions

**Figure 3:**
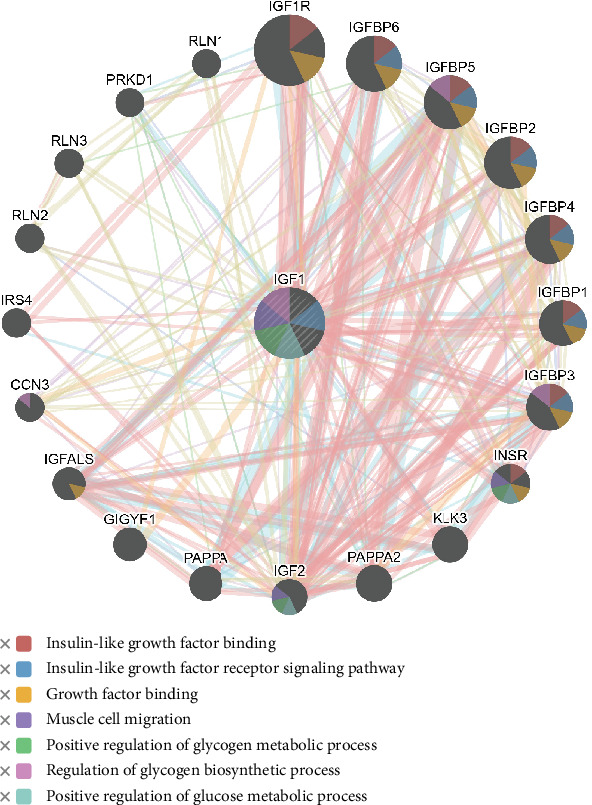
Concentric bipartite by GeneMANIA [29] illustrates gene–gene interaction networks of the IGF gene.

The GeneMANIA server has predicted the functional gene–gene interaction network for IGF and VIP genes, as depicted in Figure 3. The results indicated that IGF has physical and genetic interactions with Insulin-Like Growth Factor–Binding Protein 2 (IGFBP2) and shows coexpression primarily with IGFBP4, Protein Kinase D1 (PRKD1), insulin-like growth factor–binding protein acid labile subunit (IGFALS), Relaxin 2 (RLN2), IGFBP3, and IGF2, as shown in Figure 3.

**Figure 4:**
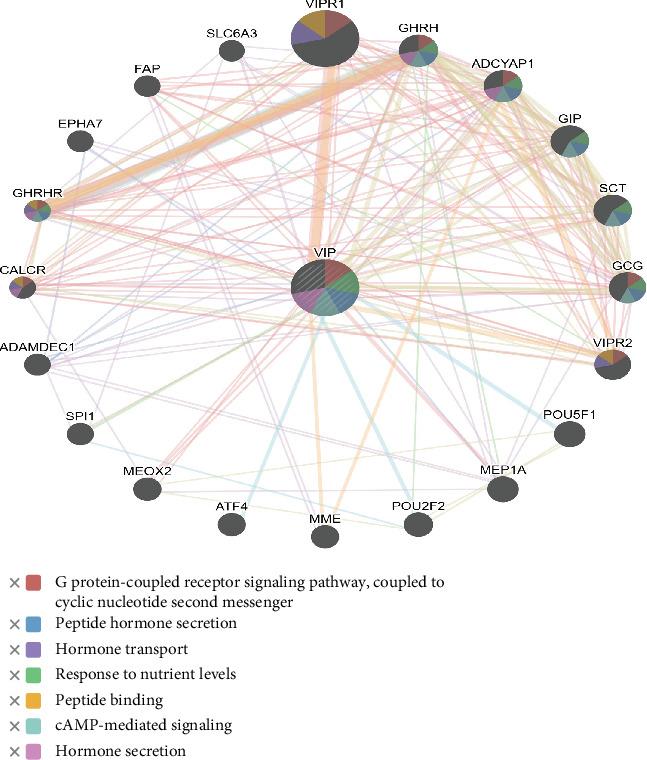
Concentric bipartite created by GeneMANIA represents gene–gene interaction networks of the VIP gene.

Similarly, the *VIP* gene interacts physically and genetically with the transcription factor PU.1 (SPI1), and is coexpressed mostly with the calcitonin receptor (*CALCR*), Adenylate Cyclase–Activating Polypeptide 1 (*ADCYAP1*), ADAM-Like Decysin 1 (*ADAMDEC1*), Ephrin Type A receptor 7 (*EPHA7*), GH-releasing hormone (*GHRH*), Solute Carrier Family 6 Member 3 (*SLC6A3*), gastric inhibitory polypeptide (*GIP*), and glucagon (*GCG*) (Figure 4).

## 3. Discussion

In mammals, IGF and VIP genes are evolutionarily conserved peptides that function as endocrine hormones and autocrine/paracrine factors. They are extensively present in the central and peripheral nervous systems, as well as in the digestive, respiratory, reproductive, and cardiovascular systems, serving as neurotransmitters and neuroendocrine release factors [30]. Given their widespread expression in mammalian tissues, a deep understanding of their regulatory mechanisms is essential. In this study, we employed various online bioinformatics tools to perform in silico analysis of promoter and gene body sequences of 15 IGF and VIP genes related to reproductive traits in chickens, aiming to identify characteristics that may serve as exemplary for other avian species. Furthermore, the results of our analysis provided vital information on the upstream regulatory elements and their corresponding TFs involved in regulatory mechanisms that influence the IGF and VIP genes during reproduction and embryo development in chickens.

TSS identification is a crucial step in gene expression and occurs at various promoter positions with different efficiencies [31]. In the present in silico analysis, the majority of the promoter sequences (73.33%) had multiple TSSs. This result is in agreement with Samuel and Dinka [32] who reported the presence of multiple TSSs in olfactory receptor genes in cattle, whereas it is contrary to Abera and Dinka [33] who reported the presence of single transcription sites in the MAGE gene encoding for embryonic development in cattle. This indicates the presence of alternative promoters of the genes, and it contributes to transcript isoform diversity in mammals.

The current study also revealed that approximately half of the examined TSSs (46.7%) are located at a distance below −500 bp from the start codon. This result is in agreement with Mu et al. [34] who reported a TSS location of −515 bp for the ovine *DKK1* gene, Abera and Dinka [33] who reported 42.1% TSS location below –500 bp for the *MAGE* gene, and Pokhriyal et al. [35] who reported TSS locations at 235, 156, and 92 bp for BICP0, BICP4, and BICP22 in bovine genes, respectively.

The present analysis uncovers multiple binding motifs for IGF and VIP genes, which is significant to find all possible binding motifs including the cofactor binding motifs [36]. The majority of the discovered candidate motifs are located and distributed between –800 and –200 bp with reference to the TSS region. This is in line with Halees et al. [37] who stated that the majority of motifs are positioned proximately upstream of a TSS. The candidate motifs were equally distributed in the positive and negative strands.

The present analysis revealed that the best common motif, Motif II, bears resemblance to three major transcription factor families: zinc finger family, homeobox transcription factors, and high-mobility group domain factors, accounting for about 79.17% of the candidate transcription factors. This study found that 62.5% of the identified transcription factors interacted with the Wnt signalling pathway to coregulate transcription. This is in agreement with reports of Wang et al. [38] who described the high-mobility group transcription factors as mediators of the Wnt signalling pathway and Yu et al. [39] who reported homeobox transcription factors as coregulators. The Wnt signalling pathways have critical roles in axis patterning, cell fate specification, cell proliferation, and cell migration [40].

The current findings revealed that CPEB1, MAFB, SOX15, TCF7L2, TCF3, and TCF7 transcription factors had dual regulatory functions and have a crucial role in embryonic and nervous system development. Hrckulak et al. [41] reported that T-cell factor/lymphoid enhancer–binding factor proteins are the main downstream effectors of the Wnt signalling pathway. Furthermore, transcription factor CREB-1 is highly involved in the control of ovarian cell proliferation and steroidogenesis [42]. Evidence suggested that transcription factors like TCF and LEF play key regulatory functions in mammalian cells [43–45]. The current study also shows that Bcl11B transcription factors repress transcription and play key roles in normal T-cell development, as we revealed from the UniProt database. In agreement with this result, Lennon et al. [46] have shown the fundamental roles of Bcl11B in fetal development.

Studies identified that CGIs are highly involved in dual-mode gene regulatory processes in higher eukaryotic species [47]. In this study, IGF and VIP genes associated with chicken reproductive traits have very rich CGIs in their promoter and gene body regions. In agreement with this finding, Vinoth et al. [48] and Ummah et al. [49] found rich CpG islands in the HSP90 gene promoter region in chickens. GC content in genes is significantly associated with gene expression patterns and could be one of the important regulatory factors in the chicken genome [50].

The in silico analysis results also revealed very rich CpG islands in IGF and VIP genes, which is in agreement with the first method, CpG-Profile algorithm. A similar finding was reported by Abera and Dinka [33] who indicated slightly rich CpG islands for MAGE genes encoding for embryonic development in cattle. However, Wagari et al. [51] reported poor CGIs using *MspI* enzyme digestion for sheep KAP genes. CpG islands are frequently linked with the promoters of most housekeeping genes and numerous tissue-specific genes, thereby playing significant regulatory roles and serving as markers for genes [52].

It has been reported that genes containing tandem repeats in their promoters exhibit higher rates of transcriptional divergence [53], and it indicates a greater tendency for mutation [54]. The current study revealed that many tandem repeats were identified in 10 gene promoters out of 15, suggesting that they have an increased potential for accumulation of mutations during replication. The presence of tandem repeats in these promoters can be used for mutational study, and it might also participate in gene expression regulation.

The gene–gene interaction network revealed that approximately 77.64% of genes show physical interactions and 8.01% of genes show coexpression in both IGF and VIP networks. The 20 most frequently altered genes were involved in the IGF network, including pregnancy-associated plasma protein A2 (*PAPPA2*), *RLN2*, and Kallikrein-Related Peptidase 3 (KLK3), which has a key role in regulating biological functions in reproduction. Guo et al. [1] reported that the IGF system is highly conserved and is involved in the regulation of egg production, growth, and carcass traits in chickens. Similarly, *VIP* genes interact with important traits such as *CALCR*, *ADCYAP1*, *ADAMDEC1*, *EPHA7*, *GHRH*, *SLC6A3*, and *GCG.* Based on the analysis of interaction patterns, we may understand that the IGF and VIP genes might affect the function of the related genes, which highlights the importance of these linked and coexpressed genes in complex traits like reproduction.

In conclusion, the current findings showed that regulatory elements found in the promoter region of IGF and VIP genes may play important roles in the chicken reproductive traits and coregulate transcription through interacting with the Wnt pathways. Understanding these gene regulatory elements in the promoter regions could be beneficial in the poultry industry to improve reproductive performances. However, further experimental studies are needed to validate the potential role of identified transcription factors and their motifs in regulating IGF and VIP genes in chickens. Thus, the findings should be essentially validated using experimental studies such as gene expression assays.

## 4. Materials and Methods

### 4.1. Retrieval of the IGF and VIP Genes

The IGF and VIP gene sequences were retrieved from the NCBI database via a web server (https://www.ncbi.nlm.nih.gov (accessed on January 22, 2024). In this analysis, a total of 15 functional, protein-coding genes that have a coding sequence (CDS) and start codon at the beginning of the sequence were considered (Table 8). Full-length sequences of these genes, as well as their CDSs, were retrieved. All gene sequences were cross-checked with the UniProtKB database (https://www.uniprot.org).

### 4.2. Determination of Transcription Start Sites and Promoter Regions

In order to determine TSSs, we extracted 1 kb sequences upstream of the start codon from each gene [55]. The Neural Network Promoter Prediction (NNPP Version 2.2) tool was used to determine the TSSs by setting the minimum standard predictive score (between 0 and 1) with a cut-off value of 0.8 [56]. This tool has the ability to pinpoint precisely the position of a TSS for a given gene. The TSS with the highest prediction score was considered statistically significant and accurate for sequences containing multiple TSSs. In addition, we excised 1 kb sequences upstream of each TSS to determine the promoter regions, as previously described by Michaloski et al. [57] for mouse odorant and vomeronasal receptor (V1R) gene.

### 4.3. Identification of Common Candidate Motifs and Transcription Factors

To discover common candidate motifs, the predicted promoter sequences of IGF and VIP genes were analysed using the MEME Version 5.5.4 searches [58]. Then, MEME searches for statistically significant candidate motifs in the input sequence set. Buttons on the MEME HTML output allow one or all of the candidate motifs to be forwarded for further analysis by TOMTOM [59] for TF. The output of TOMTOM includes LOGOS representing the alignment of the candidate motif and TF with the *p* value and *q*-value (a measure of false discovery rate) of the match and links back to the parent transcription database for more detailed information about it [28].

### 4.4. Search for CpG Islands and Tandem Repeats

A 2 kb sequence upstream of the start codon was analyzed for each gene to detect tandem repeats and CpG islands. GC-Profiles 2.0 software was used to search for CpG islands with the search criteria: GC content ≥ 50%, Obs CpG/Exp CpG ≥ 0.6, and length ≥ 200 bp [60]. The MISA-web search tool [61] was used to search tandem repeats in the promoter and coding regions of each gene. Furthermore, *MspI* restriction enzyme cutting sites were searched using CLC Genomics Workbench Version 23.0.5, with fragment sizes ranging from 40 to 220 bps. This search is important for the detection of CGIs, and it recognizes CCGG sites [62].

### 4.5. Analysis of Gene–Gene Interactions

The gene–gene interaction network of the IGF and VIP gene was predicted by GeneMANIA (http://www.genemania.org). It predicts gene function and generates information such as gene interaction, coexpression, colocalization, shared protein domains, and pathways involved [29].

## Figures and Tables

**Table 1:** TSS number and predictive score value for IGF and VIP genes associated with chicken reproductive traits.

**Gene name**	**Corresponding promoter region name**	**No. of TSSs identified**	**Predictive score value**	**Distance of best TSSs from ATG**
IGF1	Pro-IGF1	2	0.80, 0.85	−1793
IGF1R	Pro-IGF1R	3	0.84, 0.86, 0.99	−763
IGF2	Pro-IGF2	1	0.98	−314
IGF2BP1	Pro-IGF2BP1	8	0.83, 0.84, 0.86,0.89, 0.91, 0.92, 0.96, 0.99	−162
IGF2BP2	Pro-IGF2BP2	1	0.82	−124
IGF2BP3	Pro-IGF2BP3	3	0.83, 0.83, 0.92	−792
IGFBP1	Pro-IGFBP1	2	0.91, 0.99	−795
IGFBP2	Pro-IGFBP2	2	0.85, 0.88	−4626
IGFBP3	Pro-IGFBP3	4	0.85, 0.85, 0.86, 0.97	−694
IGFBP4	Pro-IGFBP4	2	0.82, 1.00	−41
IGFBP5	Pro-IGFBP5	1	0.86	−102
IGFBP7	Pro-IGFBP7	1	0.82, 0.92	−3240
VIP	Pro-VIP	4	0.86, 0.93, 0.94, 1.00	−458
VIPR1	Pro-VIPR1	6	0.83, 0.89, 0.97, 0.98, 1.00, 1.00	−493
VIPR2	Pro-VIPR2	3	0.81, 0.87, 0.97	−708

**Table 2:** Identified common candidate motifs in promoter regions of IGF and VIP genes associated with chicken reproductive traits.

**Discovered candidate motif**	**Number (%) of promoters containing each one of the motifs**	**E** ** value**	**Motif width**
I	11 (73.3)	5.8e − 055	41
II	12 (80.0)	3.7e − 021	29
III	11 (73.3)	1.6e − 019	49
IV	6 (40.0)	6.1e − 010	49
V	7 (46. 7)	3.8e − 005	39

**Table 3:** The list of TF candidates which could bind to Motif II.

**TF family**	**Regulatory elements**	**Regulatory mode**	**Function**	**Major tissue expression**
Zinc finger factors	ZNF384 (*Homo sapiens*)	Activation	Regulates translation initiation during oocyte maturation and early development	Uterus and ovary
	Tfap2e (*Mus musculus*)	Activation	Play a role in the development and differentiation of cells	Primary oocyte
	Mtf1 (*Mus musculus*)	Activation	Regulates the expression of metalloproteases in response to intracellular zinc and functions as a catabolic regulator of cartilages	Bone marrow
	Zfp105 (*Mus musculus*)	Activation	It is male germ cell factor and plays a role in male reproduction	Ovary and testis
	CPEB1 (*Homo sapiens*)	Dual	Regulates translation initiation during oocyte maturation and early development	Ovary, brain, and heart
	Bcl11B (*Mus musculus*)	Repressor	Key regulator of both differentiation and survival of T lymphocytes during thymocyte development in mammals	Brain and thymus

Homeo-box transcription factors^a^	ONECUT1 (*Homo sapiens*)	Activation	Important for liver gene transcription	Liver and testis
	ONECUT3 (*Homo sapiens*)	Activation	Regulation of beta-cell development and nervous system development	Testis and retina
	CDX2 (*Homo sapiens*)	Activation	It plays a central role from early differentiation to maintenance of the intestinal epithelial	Colon and small intestine
	Hnf1A (*Mus musculus*)	Activation	Regulates the tissue-specific expression of multiple genes, especially in pancreatic islet cells and in liver	Liver and kidney

High-mobility group domain factors^a^	LEF1 (*Homo sapiens*)	Activation	Participates in the Wnt signalling pathway	Thymus and oocyte
	SOX10 (*Homo sapiens*)	Activation	It plays a central role in the development and maturation of nerve cells in the brain	Brain, heart, and small intestine
	TCF7L2 (*Homo sapiens*)	Dual	Participates in the Wnt signalling pathway	Lateral nuclear group of thalamus
	TCF7L1 (*Homo sapiens*)	Activation	Participates in the Wnt signaling pathway	Ovary and skin keratinocytes
	Tcf3 (*Mus musculus*)	Dual	Proteins play major roles in determining tissue-specific cell fate during embryogenesis	Ovary and skin keratinocytes
	Lef1 (*Homo sapiens*)	Activation	Participates in the Wnt signaling pathway	Embryonic postanal tail
	Tcf7 (*Mus musculus*)	Dual	Required for the development of natural killer receptor–positive lymphoid tissue inducer T cells	Oocyte and thymus
	SOX15 (*Homo sapiens*)	Dual	Plays a role in the development of myogenic precursor cells	Brain and testis
	SOX11 (*Mus musculus*)	Activator	Plays a redundant role with SOX4 and SOX12 in cell survival of developing tissues thereby contributing to organogenesis	Primarily in the brain and heart

MADS-box factors	Srf (*Mus musculus*)	Activator	It controls expression of genes regulating the cytoskeleton during development, morphogenesis, and cell migration	Embryonic postanal tail

NFAT factors^a^	Nfatc1 (*Mus musculus*)	Activator	It controls gene expression in embryonic cardiac cells	Spleen and thymus

MAF factors^a^	Mafb (*Mus musculus*)	Dual	Plays a pivotal role in regulating lineage-specific hematopoiesis	Kidney, gut, lung, and brain

Nuclear transcription factors^a^	Foxa2 (*Mus musculus*)	Activator	Plays a key role in embryonic development and establishment of tissue-specific gene expression	Lung, liver, and uterus

EST transcription factors	SPIB (*Homo sapiens*)	Activator	Promotes development of plasmacytoid dendritic cells	Lymph node

*Note:* ZNF384—zinc finger protein 384; Tfap2e—transcription factor AP-2 epsilon; Mtf1—metal-regulatory transcription factor 1; Zfp105—zinc finger protein 105; CPEB1—cytoplasmic polyadenylation element–binding protein 1; Bcl11B—B-cell lymphoma/leukaemia 11B; ONECUT1—one cut homeobox 1; ONECUT3—one cut homeobox 3; CDX2—caudal type homeobox 2; Hnf1A—hepatocyte nuclear factor-1 alpha; LEF1—lymphoid enhancer binding factor 1; SOX10—SRY-box transcription factor 10; TCF7L2—transcription factor 7–like 2; TCF7L1—transcription factor 7–like 1; Tcf3—transcription factor 3, T cell specific; Lef1—lymphoid enhancer binding factor 1; Tcf7—transcription factor 7, T cell specific; SOX15—SRY-box transcription factor 15; SOX11—SRY-box transcription factor 11; Srf—serum response factor; Nfatc1—nuclear factor of activated T cells; Mafb—musculoaponeurotic fibrosarcoma oncogene homolog B; Foxa2—forkhead box protein A2; SPIB—Spi-B transcription factor.

^a^Transcription factor family regulating the Wnt pathway.

**Table 4:** CpG islands identified in upstream and gene body regions for chicken IGF and VIP genes.

	**Promoter region**							**Gene body region**						
	**CGI_No.**	**Start**	**End**	**Length (bp)**	**CpGo/e**	**GC%**	**CpG%**	**CGI_No.**	**Start**	**End**	**Length (bp)**	**CpGo/e**	**GC%**	**CpG%**
IGF1	0	—	—	—	—	—	—	—	—	—	—	—	—	—
IGF1R	1	602	2000	1399	1.12	70.62	13.94	1	1	425	425	0.92	54.35	6.82
	—	—	—	—	—	—	—	2	3413	3672	260	0.74	50.38	4.62
IGF2	0	—	—	—	—	—	—	1	195	564	370	0.60	64.32	6.22
IGF2BP1	1	833	2000	1168	0.80	64.47	8.30	1	1	315	315	0.71	51.75	4.76
	—	—	—	—	—	—	—	2	1122	1382	261	0.60	60.92	5.36
IGF2BP2	1	833	2000	1168	0.80	64.47	8.30	1	1	453	453	0.74	57.84	6.18
	—	—	—	—	—	—	—	2	1561	1768	208	0.80	53.85	5.77
IGF2BP3	1	8	445	438	0.60	59.13	5.25	—	—	—	—	—	—	—
	2	1662	2000	339	0.88	82.60	15.04	—	—	—	—	—	—	—
IGFBP1	0	—	—	—	—	—	—	1	1	388	388	0.75	71.91	9.54
IGFBP2	1	376	932	557	0.60	57.81	5.03	1	1	936	936	0.80	69.12	9.51
IGFBP3	1	847	1344	498	0.78	57.43	6.43	1	1	370	370	0.75	71.91	9.54
	2	1670	2000	331	0.96	77.95	14.50	—	—	—	—	—	—	—
IGFBP4	1	136	1421	1286	0.60	69.05	7.15	1	1	783	783	0.85	71.14	10.73
	2	1672	2000	329	0.97	82.98	16.41	—	—	—	—	—	—	—
IGFBP5	1	1491	1856	366	0.60	59.29	4.92	1	1	810	810	0.76	66.54	8.40
IGFBP7	0	—	—	—	—	—	—	1	1	553	553	1.10	80.29	17.54
VIP	1	1	2000	2000	0.80	56.30	6.35	—	—	—	—	—	—	—
VIPR1	1	1757	2000	244	1.13	75.82	15.98	1	1	273	273	0.60	58.24	5.13
VIPR2	1	1258	2000	743	0.84	67.56	9.42	—	—	—	—	—	—	—

**Table 5:** Lists of tandem repeats identified in the promoter and gene body regions for chicken IGF and VIP genes.

**Gene name**	**Promoter region**						**Gene body region**					
	**TR nr.**	**TR type**	**TR sequence**	**Size**	**Start**	**End**	**TR nr.**	**TR type**	**TR sequence**	**Size**	**Start**	**End**
IGF1	1	p2	(GA)6	12	1726	1737						
IGF1R	1	p3	(GCG)7	21	52	72						
IGF2BP1	1	p4	(CCGC)5	20	960	979						
IGF2BP3	1	p1	(C)11	11	422	432						
IGF2BP3	2	p2	(CT)11	22	907	928	1	p3	(CTG)5	15	1178	1192
VIP	1	p1	(A)12	12	1	12						
VIPR1	1	p1	(A)10	10	632	641						
IGFBP2	1	p1	(T)10	10	934	943						
IGFBP2	2	p1	(T)11	11	4743	4753						
IGFBP2	3	p1	(T)12	12	4916	4927						
IGFBP3	1	p1	(T)12	12	530	541						
IGFBP4	1	p1	(C)10	10	216	225	1	p3	(GGC)11	33	2	59
IGFBP4	2	p1	(G)11	11	741	751						
IGFBP4	3	p3	(GCC)5	15	938	952						
IGFBP7	1	p1	(T)13	13	3796	3808						

*Note:* p1—mononucleotide repeat; p2—dinucleotide repeats; p3—trinucleotide repeats; p4—tetranucleotide repeats.

Abbreviation: TR—tandem repeat.

**Table 6:** *MspI* cutting sites and fragment sizes in promoter region for chicken IGF and VIP gene sequences.

**Sequence name**	**Promoter region**	
	**No. and positions of *MspI* cutting sites**	**Fragment sizes (between 40 and 220 bps)**
IGF1	No cut	—
IGF1R	14 (9, 151, 172, 252, 274, 325, 342, 528, 560, 683, 709, 790, 810, 964)	142, 80, 51, 186, 123, 81, 154
IGF2	No cut	—
IGF2BP1	15 (9, 33, 85, 195, 452, 465, 472, 556, 574, 585, 619, 862, 925, 946, 989)	52, 110, 84, 63, 43
IGF2BP2	7 (753, 808, 844, 882, 922, 965, 972)	55, 40,43
IGF2BP3	15 (122, 167, 172, 177, 284, 324, 384, 434, 462, 521, 672, 758, 764, 792, 993)	45, 107, 40, 60, 50, 59, 151, 86, 201
IGFBP1	No cut	—
IGFBP2	4 (160, 3682, 3710, 3714)	—
IGFBP3	9 (27, 289, 670, 764, 893, 910, 953, 976, 992)	94, 129, 43
IGFBP4	9 (52, 142, 273, 284, 795, 833, 855, 886, 895)	90, 131
IGFBP5	2 (912, 952)	40
IGFBP7	2 (3272, 3969)	—
VIP	2 (272, 969)	—
VIPR1	1 (766)	—
VIPR2	7 (420, 446, 609, 790, 817, 924, 985)	163, 181, 107, 61

**Table 7:** *MspI* cutting sites and fragment sizes in gene body region for chicken IGF and VIP gene sequences.

**Sequence name**	**Gene body region**	
	**No. and positions of *MspI* cutting sites**	**Fragment sizes (between 40 and 220 bps)**
IGF1	1 (143)	—
IGF1R	10 (753, 2151, 2693, 3079, 3087, 3501, 3544, 3817, 3865, 3979)	43, 48, 114
IGF2	2 (435, 548)	113
IGF2BP1	11 (43, 110, 240, 384, 531, 1172, 1204, 1293, 1327, 1336, 1656)	67, 130, 144, 147
IGF2BP2	9 (9, 69, 104, 125, 148, 478, 559, 923, 1306)	60, 81
IGF2BP3	1 (970)	—
IGFBP1	7 (55, 158, 166, 185, 220, 294, 373)	103, 74, 79
IGFBP2	10 (97, 163, 171, 202, 220, 278, 302, 417, 685, 906)	66, 58, 115
IGFBP3	7 (14, 60, 73, 79, 166, 242, 358)	46, 87, 76, 116
IGFBP4	8 (64, 166, 201, 242, 276, 510, 661, 724)	102, 41, 151, 63
IGFBP5	9 (37, 145, 182, 405, 462, 540, 690, 696, 745)	108, 57, 78, 150, 49
IGFBP7	12 (8, 61, 107, 223, 255, 323, 350, 400, 419, 433, 466, 803)	53, 46, 116, 68, 50
VIP	No cut	—
VIPR1	2 (395, 1302)	—
VIPR2	No cut	—

**Table 8:** IGF and VIP genes retrieved on January 22, 2024, from the NCBI genome browser, used in this study.

**S/N**	**Gene name**	**Gene ID**	**Accession number**	**Chr. location**	**Genome coordinate**	**Exon count**
1	IGF1	418090	NC_052532.1	1	(55326358..55374742)	4
2	IGF1R	395889	NC_052541.1	10	(16697226..16844358)	21
3	IGF2	395097	NC_052536.1	5	(13375612..13394087)	4
4	IGF2BP1	395953	NC_052558.1	27	(3257187..3283328)	15
5	IGF2BP2	396315	NC_052538.1	7	(22879236..22951723)	17
6	IGF2BP3	420617	NC_052533.1	2	(31057514..31164938)	18
7	IGFBP1	408027	NC_052533.1	2	(54589779..54595974)	4
8	IGFBP2	396315	NC_052538.1	7	(22879236..22951723)	11
9	IGFBP3	420790	NC_052533.1	2	(54603575..54621232)	4
10	IGFBP4	374271	NC_052558.1	27	(4441013..4446307)	4
11	IGFBP5	424220	NC_052538.1	7	(22854992..22876649)	4
12	IGFBP7	422620	NC_052535.1	4	(48656657..48686876)	8
13	VIP	396323	NC_052534.1	3	(49169925..49176636)	7
14	VIPR1	395329	NC_052533.1	2	(1764256..1874747)	14
15	VIPR2	420453	NC_052533.1	2	(9615391..9670983)	14

## Data Availability

The data that support the findings of this study are available from the corresponding author upon reasonable request.
